# Inverse pH Regulation of Plant and Fungal Sucrose Transporters: A Mechanism to Regulate Competition for Sucrose at the Host/Pathogen Interface?

**DOI:** 10.1371/journal.pone.0012429

**Published:** 2010-08-26

**Authors:** Kathrin Wippel, Anke Wittek, Rainer Hedrich, Norbert Sauer

**Affiliations:** 1 Molekulare Pflanzenphysiologie, Universität Erlangen-Nürnberg, Erlangen, Germany; 2 Molekulare Pflanzenphysiologie und Biophysik, Julius-von-Sachs-Institut, Biozentrum, Universität Würzburg, Würzburg, Germany; Umeå Plant Science Centre, Sweden

## Abstract

**Background:**

Plant sucrose transporter activities were shown to respond to changes in the extracellular pH and redox status, and oxidizing compounds like glutathione (GSSG) or H_2_O_2_ were reported to effect the subcellular targeting of these proteins. We hypothesized that changes in both parameters might be used to modulate the activities of competing sucrose transporters at a plant/pathogen interface. We, therefore, compared the effects of redox-active compounds and of extracellular pH on the sucrose transporters UmSRT1 and ZmSUT1 known to compete for extracellular sucrose in the *Ustilago maydis* (corn smut)/*Zea mays* (maize) pathosystem.

**Methodology/Principal Findings:**

We present functional analyses of the *U. maydis* sucrose transporter UmSRT1 and of the plant sucrose transporters ZmSUT1 and StSUT1 in *Saccharomyces cerevisiae* or in *Xenopus laevis* oocytes in the presence of different extracellular pH-values and redox systems, and study the possible effects of these treatments on the subcellular targeting. We observed an inverse regulation of host and pathogen sucrose transporters by changes in the apoplastic pH. Under none of the conditions analyzed, we could confirm the reported effects of redox-active compounds.

**Conclusions/Significance:**

Our data suggest that changes in the extracellular pH but not of the extracellular redox status might be used to oppositely adjust the transport activities of plant and fungal sucrose transporters at the host/pathogen interface.

## Introduction

Only recently, UmSRT1, the first fungal sucrose transporter, was identified in the plasma membrane of the maize (*Zea mays*) pathogen *Ustilago maydis* (corn smut [1]). The UmSRT1 protein is a high affinity sucrose/H^+^-symporter with a substrate affinity (K_m_-value: 26 µM) that is significantly higher than that of most plant sucrose transporters [2]. Its gene is expressed exclusively in hyphae growing *in planta* suggesting that the encoded protein is specifically involved in the uptake of sucrose from the plant apoplast. In fact, deletion of the *UmSRT1* gene results in an almost complete loss of symptom development and tumor formation. This demonstrated that UmSRT1 is essential for the virulence of *U. maydis* [1].

The biotrophic basidiomycete *U. maydis* occurs ubiquitously and depends on living plant material for growth and propagation. As it does not use aggressive virulence strategies it can persist for long periods on its live host without causing induction of apparent defense responses [3], [4]. Upon plant cell infection, *U. maydis* hyphae invaginate the plasma membrane of infected cells forming a narrow contact zone, where host and pathogen are separated only by their plasma membranes and a thin interface. At later stages of development, *U. maydis* hyphae typically grow along the phloem of infected maize plants, where they have access to sucrose released from this long-distance transport tissue [1].

Within infected maize plants, *UmSRT1*-expressing *U. maydis* hyphae compete with the mays ZmSUT1 sucrose transporter for apoplastic sucrose. This maize transporter is responsible for the loading of sucrose into the maize phloem, for the retrieval of sucrose leaking out of the phloem cells, and possible also for the release of sucrose under defined conditions [5], [6].

ZmSUT1 was shown to import or release sucrose as a function of extracellular pH, transmembrane sugar gradient and voltage [6]. Moreover, a regulation of ZmSUT1 by changes in the extracellular redox potential has been proposed [7]. When the sensitivity to various redox-active compounds was tested with heterologously expressed plant sucrose transporters [*ZmSUT1* expressed in *Xenopus laevis* oocytes; potato (*Solanum tuberosum*) *StSUT1* expressed in *Saccharomyces cerevisiae*], a strong, up to 10-fold activation of transporter activities in the presence of oxidizing compounds was observed [oxidized glutathione (GSSG) or L-cystine]. In contrast, the presence of reducing compounds [reduced glutathione (GSH) or dithiothreitol (DTT)] reduced the activities [7]. Based on associated studies on SlSUT1, a sucrose transporter from tomato (*Solanum lycopersicum*), the redox activation observed with ZmSUT1 or StSUT1 was explained by improved targeting of transport proteins to the plasma membrane in the presence of oxidizing compounds (H_2_O_2_, L-cystine or GSSG [7]). This hypothesis seemed corroborated by the fact that detergent extracts from H_2_O_2_-treated plasma membranes revealed an increased content of dimerized SlSUT1 that accumulated in raft-like structures [8] of the plasma membrane.

As ZmSUT1 is active in the above-mentioned host/pathogen contact zone of *U. maydis*-infected maize plants, it was tempting to speculate that changes in the extracellular pH and/or redox status might affect not only the maize transporter ZmSUT1, but also the UmSRT1 transporter of the pathogen [1], and that the two transporters might even be affected in different ways.

Our analyses revealed that, in fact, the proposed modulation of host and pathogen sucrose transport activities might be obtained by changes in the extracellular pH. The different pH-dependences of UmSRT1 and plant sucrose transporters might represent a mechanism for inverse regulation of plant and fungal transporters at the host/pathogen interface. Detailed analyses of the effects of redox-active compounds on the transport activity of the *U. maydis* sucrose transporter UmSRT1, however, did neither observe redox regulation for UmSRT1, nor could the reported redox-sensitivity of the plant sucrose transporters ZmSUT1 and StSUT1 be confirmed. Moreover, the targeting of UmSRT1 to the yeast plasma membrane was not affected by redox-active compounds.

## Results

### Effect of reducing and oxidizing compounds on the *U. maydis* sucrose transporter UmSRT1

**Figure 1:**
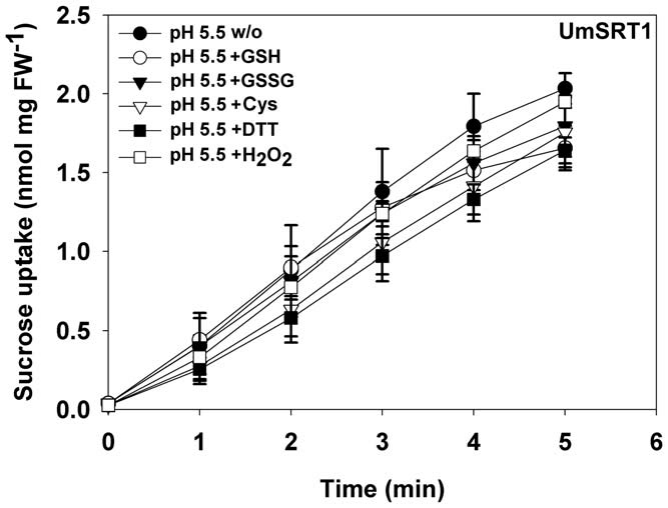
Effect of different redox reagents on the UmSRT1-mediated sucrose transport in yeast. Uptake was measured in sodium-phosphate buffer pH 5.5 in the presence of the indicated compounds. Cysteine was added to a final concentration of 5 mM, all other compounds to a final concentration of 10 mM (pH-value controlled; n = 3±SE).

The effects of different redox-active compounds on the UmSRT1 sucrose transporter were studied in yeast cells expressing an *UmSRT1* cDNA [1] from the yeast *PMA1* promoter (plasma membrane H^+^-ATPase 1). Figure 1 shows the transport rates of the *UmSRT1*-expressing strain at pH 5.5, the pH previously shown to be optimal for UmSRT1-mediated sucrose transport. Acidic pH-values of about 5.5 are likely to reflect the physiological pH in the plant apoplast. However, irrespective of the reducing or oxidizing potential of the used compound (GSH, GSSG, cysteine, DTT, H_2_O_2_) we reproducibly observed a slightly negative effect on the UmSRT1-mediated uptake of sucrose. This suggested to us that any alteration in the redox status of the yeast cell wall reduces the transport capacity of the fungal UmSRT1 protein, a clear difference to what has been shown for plant sucrose transporters [7]. This suggested that changes in the extracellular redox status might have opposite effects on host and pathogen sucrose transporters.

### Reducing and oxidizing compounds reduce the activity of the potato sucrose transporter StSUT1

As a positive control we measured the redox regulation of a plant sucrose transporter in yeast cells expressing the cDNA of the potato sucrose transporter StSUT1 [9]. Experiments with StSUT1 were performed at pH 5.5 and pH 7.0. A neutral pH of 7.0 (used in [7]) does not reflect the physiological conditions in the plant apoplast and provides only a weak driving force for proton-coupled sucrose transport.

**Figure 2 pone-0012429-g002:**
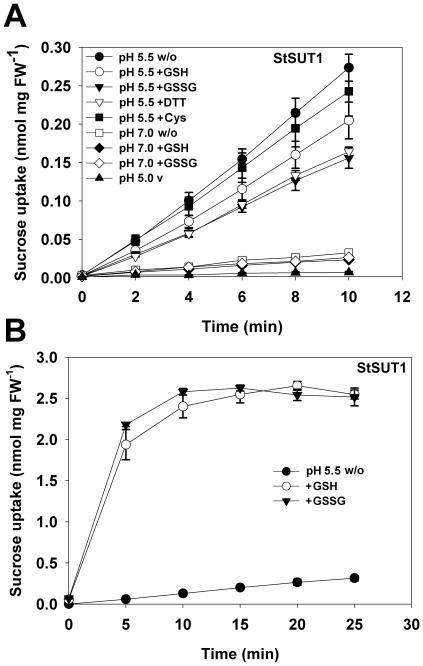
Effect of different redox reagents on the StSUT1-mediated sucrose transport in yeast. A: Uptake was measured in sodium-phosphate buffer pH 5.5 or pH 7.0 in the presence of the indicated compounds. Cysteine was added to a final concentration of 5 mM, all other compounds to a final concentration of 10 mM. pH 5.5 v  =  vector control. (pH-value controlled; n = 3 ± SD). B: Uptake was measured in sodium-phosphate buffer pH 5.5 in the presence of the indicated compounds that were added from unbuffered solutions. Both unbuffered GSSG (10 mM) and unbuffered GSH (10 mM) reduced the pH-value to 3.3 (n = 3±SE).

As expected, StSUT1-mediated sucrose transport rates at pH 7.0 were significantly lower than at pH 5.5 (Fig. 2A). Nevertheless, as the transport rates of the *StSUT1*-expressing strain at pH 7.0 were slightly higher than those in the vector-transformed control (Fig. 2A), we compared the effects of GSH and GSSG both at pH 5.5 and at pH 7.0. Unexpectedly, at both pH-values we were unable to detect a stimulating effect of GSH or GSSG on the transport of sucrose. We rather observed a reduction of 30% to 40% of the sucrose transport activities under both conditions with both compounds. This result was further supported by analyses with L-cysteine or DTT at the physiological pH of 5.5. Like GSSG, DTT tended to decrease StSUT1-mediated sucrose transport rates. This was essentially the same as what was obtained with the *U. maydis* transporter UmSRT1. Based on these results, we could no longer hold up our hypothesis of an opposite regulation of host- and pathogen sucrose transporters by the extracellular redox status.

Unbuffered GSH- and GSSG solutions have low pH values. If not adjusted (e.g. to pH 5.5 or pH 7.0) prior to their use for uptake analyses (Fig. 1 and 2) they will produce artifacts. When we performed StSUT1 analyses with GSH or GSSG solutions that had not been adjusted to pH 5.5 before, we obtained a massive induction of StSUT1-mediated sucrose transport with both compounds (Fig. 2B). Upon addition of GSH or GSSG to a final concentration of 10 mM, the extracellular pH decreased from 5.5 to 3.3. In our hands, only unbuffered GSH or GSSG solutions activated the transport of sucrose (Fig. 2B). Under pH-controlled conditions, however, GSH as well as GSSG inhibited the transport activity (Fig. 2A).

### Modulations of the extracellular pH have opposite effects on plant und fungal sucrose transporters

**Figure 3:**
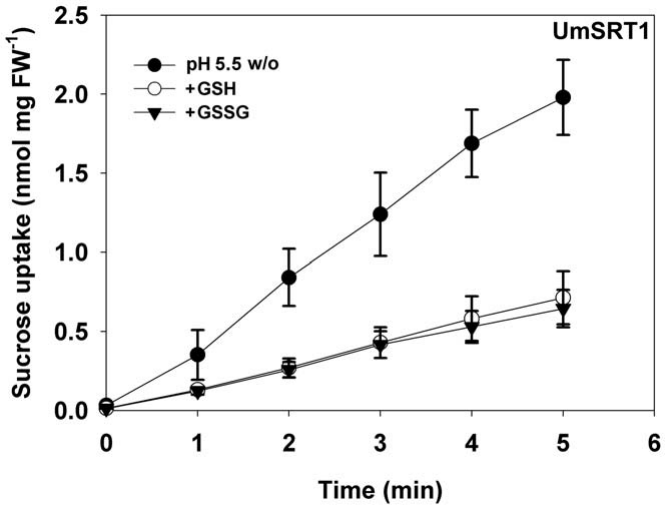
Effect of the extracellular pH on UmSRT1-mediated sucrose transport in yeast. Uptake was measured in sodium-phosphate buffer pH 5.5 in the presence of the indicated compounds that were added from unbuffered solutions. Both unbuffered GSSG (10 mM) and unbuffered GSH (10 mM) reduced the pH-values of 3.3 (n = 3±SE).

After the observation that a reduction in the extracellular pH from 5.5 to values below 3.5 was responsible for the strong activation of StSUT1-mediated sucrose transport (Fig. 2B), we tested the effects of the pH-shifts obtained with unbuffered solutions of GSH or GSSG also on UmSRT1. In contrast to StSUT1, the shifts to lower extracellular pH-values (pH 3.3) inhibited UmSRT1-dependent sucrose uptake by 60% to 70% (Fig. 3) suggesting that UmSRT1-mediated transport rates might decrease with decreasing pH values (Fig. 2B).

**Figure 4:**
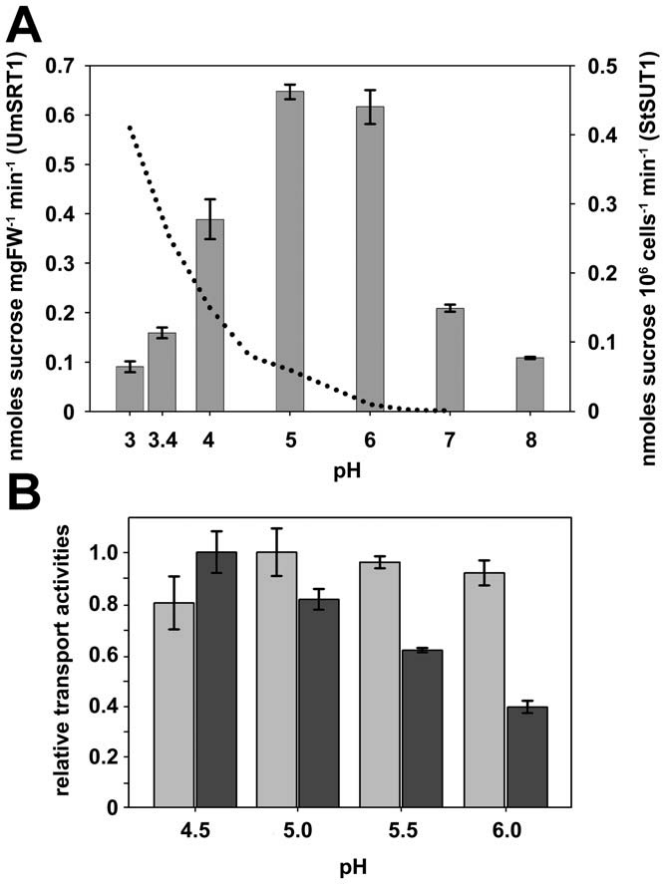
Comparison of the pH-dependences of the UmSRT1 sucrose transporter and of the plant sucrose transporters StSUT1 and ZmSUT1. A: Transport rates of UmSRT1 (bars ± SE) were measured at the indicated pH-values. Measurements from pH 5.0 to pH 8.0 were performed in 50 mM Na^+^-phosphate buffer, measurements from pH 3.0 to pH 5.0 were performed in 50 mM citrate buffer. The parallel measurements at pH 5.0 in citrate buffer and Na^+^-phosphate buffer were used to adjust the respective data (n = 3). Dotted lines show the pH dependence of the potato sucrose transporter StSUT1 (published in [10]) for comparison. B: pH-dependences of UmSRT1 (light grey bars ± SE; measured in *UmSRT1*-expressing yeast cells) and ZmSUT1 (dark grey bars ± SE; measured in *Xenopus* oocytes injected with *ZmSUT1*cRNA; membrane potential: −100 mV) were determined at the indicated pH values. The pH value yielding the highest transport rate was normalized to 1 (pH 5.0 for UmSRT1; pH 4.5 for ZmSUT1; n≥3).

This was confirmed in analyses of the pH-optimum of UmSRT1 between pH 3.0 and pH 8.0. UmSRT1 has a narrow pH-optimum at pH 5 and 6 that declines steeply at lower and higher pH-values (Fig. 4A). This pH-dependence of UmSRT1 is completely different from that of plant sucrose transporters, which respond to decreasing pH-values with a continuous increase of their transport activity. This is reflected by the increased transport rates of StSUT1 at pH 3.3 (Fig. 2B). The recently published pH-dependence of the potato sucrose transporter StSUT1 [10] is included as dotted line in Fig. 4A.

In additional and more detailed analyses we compared the pH-dependences of UmSRT1 and ZmSUT1 in the range between pH 4.5 and pH 6.0. In contrast to the transporters studied in Fig. 4A (UmSRT1 and StSUT1) these transporters compete for sucrose in *U. maydis*-infected maize plants. The chosen proton concentrations are regarded as the physiological range of apoplastic pH values. As in Fig. 4A, this analysis revealed a rather constant activity of UmSRT1 between pH 5.0 and pH 6.0 while a decline is apparent at pH 4.5. In contrast to UmSRT1, but in agreement with the data shown for StSUT1 in [10] and in Fig. 4A, the transport rates of ZmSUT1 increased steadily from pH 6.0 to pH 4.5. This demonstrates that a reduction of the extracellular pH from 6.0 to 4.5 causes a slight reduction of UmSRT1-dependent transport but a significant, almost triple increase in ZmSUT1-driven sucrose uptake.

### “Redox-regulation” is independent of the yeast glutathione transporter Hgt1p

Our data (Fig. 1 and 2A) suggest an unspecific and slightly inhibitory effect of an altered extracellular redox status on transport processes across yeast plasma membranes. Such effects are not unexpected for synthetic compounds with a strong capacity to reduce protein disulfide bonds, such as DTT (Fig. 1 and 2A). In the case of the naturally occurring compounds GSH, GSSH, or L-cysteine, however, the observed inhibitions might also be explained by the activity of the yeast glutathione transporter Hgt1p or by the activity of one of the different yeast amino acid transporters. All of these transporters mediate H^+^-symport, and the simultaneous presence of sucrose and a substrate for one of these transporters might reduce the *proton-motive force* (*pmf*) available to drive sucrose uptake. As yeast cells have several amino acids transporters but only one glutathione transporter we focused our analyses on Hgt1p.

Hgt1p has been characterized as an H^+^-symporter that can transport GSH, GSSG and different glutathione conjugates [11]. Moreover, the *HGT1* gene is constitutively expressed to a certain extent under all growth conditions and this expression is further enhanced, if glutathione is absent from the medium [11]. The simultaneous presence of high concentrations of GSH or GSSG and of [^14^C]-sucrose during transport analyses might, therefore, result in a competition of the endogenous Hgt1p transporter and the foreign sucrose transporter (UmSRT1 or StSUT1) for *pmf*.

**Figure 5:**
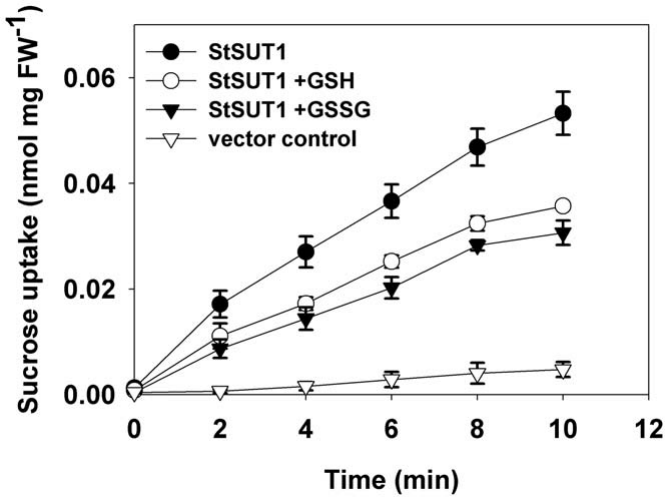
Effect of GSH and GSSG on the StSUT1-mediated sucrose transport in a Δ*hgt1* deletion mutation. Uptake of ^14^C-sucrose was measured in sodium-phosphate buffer pH 5.5 in the presence of 10 mM GSG or GSSG. The transport rates in the presence of these compounds are identical to those in Fig. 2 in an Hgt1 wild type strain (pH-values controlled; n = 3±SE).

Figure 5 shows transport analyses with an *StSUT1*-expressing yeast strain that were performed in the background of a Δ*hgt1* deletion mutation. The inhibitions (roughly 40%) by GSH and GSSG were identical to those observed in cells with an intact *HGT1* gene suggesting that the presence of the Hgt1p transporter in the yeast plasma membrane does not reduce the *pmf* available for StSUT1-mediated sucrose transport.

### GSH is not spontaneously converted into GSSG at pH 5.5

**Figure 6:**
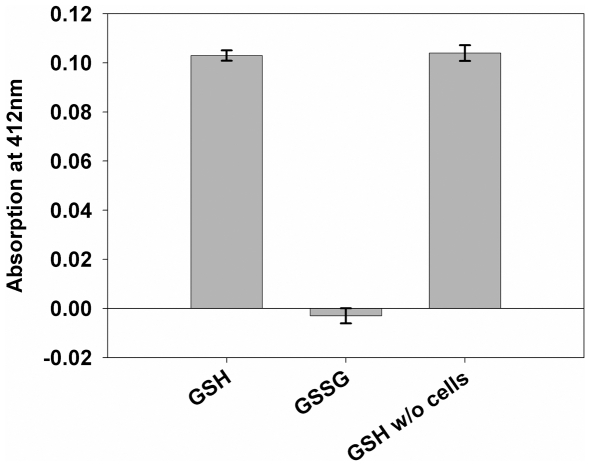
Analysis of the stability of GSH at pH 5.5 at 29°C in the presence of yeast cells with Ellman's reagent (DTNB). The amount of GSH was determined with Ellman's reagent after incubation of 10-mM GSH or 10-mM GSSG with yeast cells for 10 min (conditions of the uptake experiments shown in Figures 1, 2 and 4) and compared with the GSH levels measured after mixing the solutions at RT without cells and no further incubation. The data show that the amount of GSH is not significantly reduced during the transport tests (i.e. no GSSG is formed) and also that no GSH is formed from GSSG during the transport analyses (n = 3±SE).

Concerning the uptake studies performed at pH 5.5, we tested whether or not GSH is spontaneously oxidized during the experiment. We, therefore, determined the amount of GSH with the Ellman's reagent 5,5′-dithiobis(2-nitrobenzoic acid) (DTNB). In the presence of GSH, DTNB is reduced to 2-nitro-5-thiobencoic acid, a yellowish compound that can be quantified photometrically at 412 nm. Figure 6 shows analyses of the GSH content in a freshly made 10-mM GSH solution with a pH of 5.5 (right bar; GSH w/o cells) or in 10-mM solutions of GSH or GSSG incubated in the presence of *StSUT1*-expressing yeast cells at 29°C for 10 min. The results demonstrate that throughout the experiment neither the elevated temperature, nor the presence of yeast cells, nor the acidic pH affected GSH or GSSG stability significantly.

### Redox-active compounds do not affect the electrical properties of ZmSUT1

**Figure 7:**
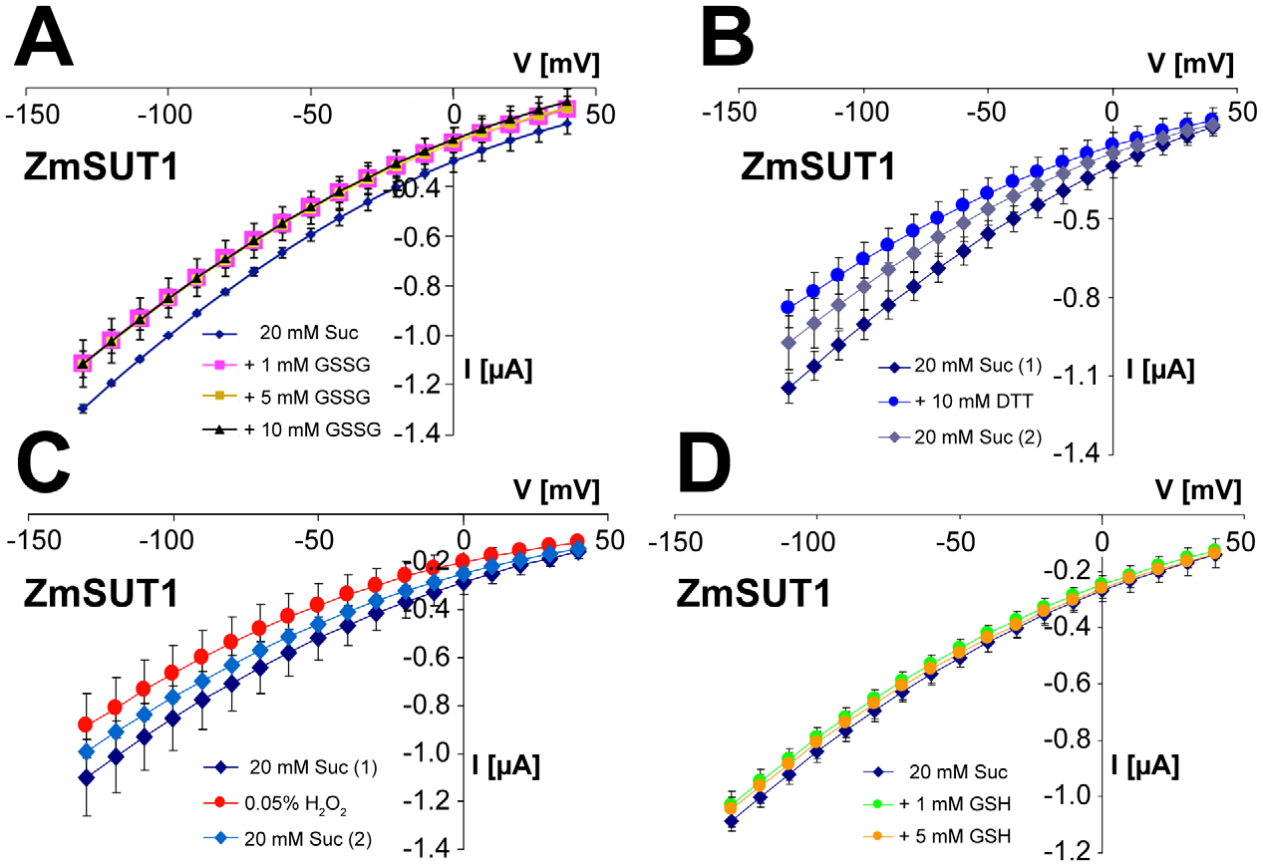
Effect of different redox reagents on sucrose-induced currents in *ZmSUT1*-expressing *Xenopus* oocytes. A: Currents elicited by 20-mM sucrose in the presence of 0-mM, 1-mM, 5-mM or 10-mM GSSG. B: Currents elicited by 20-mM sucrose in the absence of DTT before [Suc (1)] and after [Suc (2)] a measurement in the presence of 10-mM DTT. C: Currents elicited by 20-mM sucrose in the absence of H_2_O_2_ before [Suc (1)] and after [Suc (2)] a measurement in the presence of 0.05% (26.3-mM) H_2_O_2_. D: Currents elicited by 20-mM sucrose in the presence of 0-mM, 1-mM, 5-mM or 10-mM GSH. Measurements were performed at pH 5.5 at a holding potential of −70 mV in the presence of the indicated compounds (A: n = 6±SD; B: n = 10±SD; C: n = 4±SD; D: n = 3±SD).

To study whether redox reagents affect the electrogenic properties of the maize sucrose transporter ZmSUT1, we analyzed its redox sensitivity in *Xenopus laevis* oocytes. Figure 7 shows detailed analyses of the effects of GSSG (Fig. 7A), DTT (Fig. 7B), H_2_O_2_ (Fig. 7C) and GSH (Fig. 7D) under pH-controlled conditions. Similar to the response of StSUT1 in yeast, addition of extracellular reducing or oxidizing compounds ZmSUT1 in *Xenopus* oocytes showed small reductions in sucrose-coupled proton current. In more than 20 experiments we never observed a GSSG associated increase in proton fluxes. As with StSUT1, addition of unbuffered GSH or GSSG reduced the extracellular pH to values of about 3.5, which resulted in an activation of ZmSUT1 activity (not shown).

### Redox-active compounds do not alter the distribution of UmSRT1 within the plasma membrane

**Figure 8:**
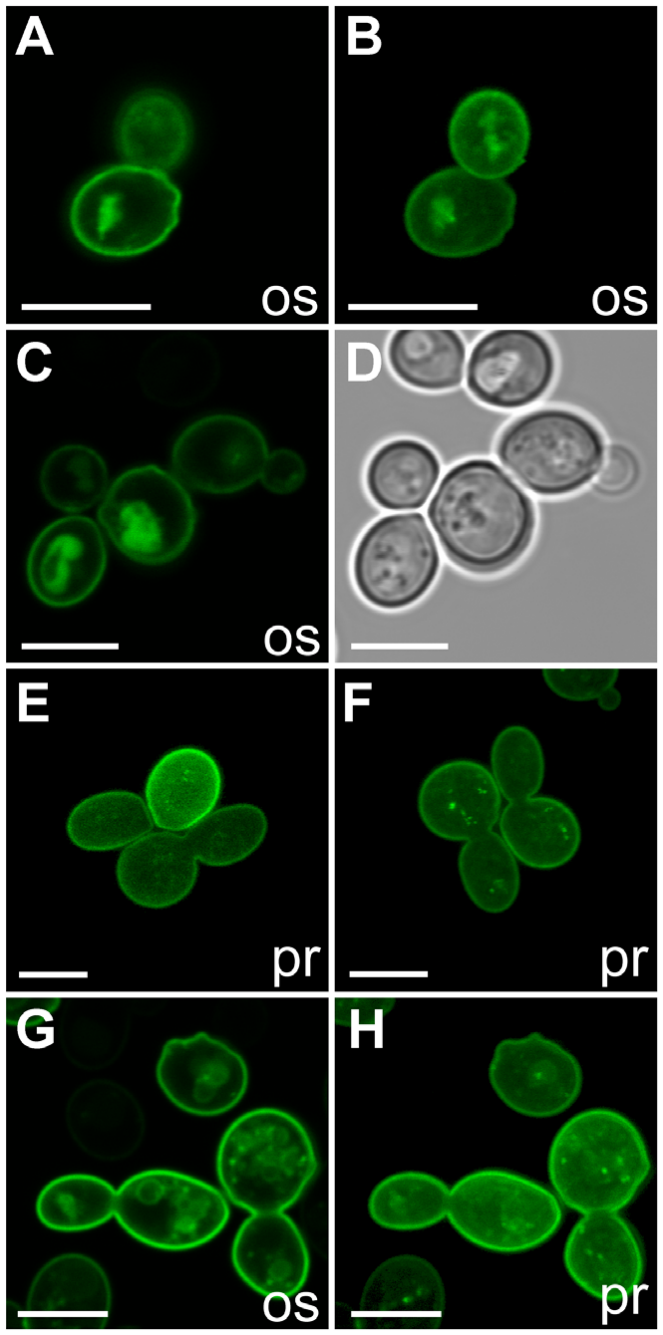
Redox-active compounds do not affect plasma membrane targeting of UmSRT1-GFP in baker's yeast. A: Optical section through cells without addition of a redox-active compound. B: Projection of several sections through the same cells as in A. C: Optical section through GSH-treated cells (GSH buffered). D: White-light image of the cells shown in C. E: Projection of several sections through GSSG-treated cells (GSSG unbuffered). F: Projection of several sections through GSH-treated cells (GSH unbuffered). G: Optical section through H_2_O_2_-treated cells. H: Projection of several sections through the H_2_O_2_-treated cells shown in G. Experiments were performed at an initial pH of 5.5 (25-mM Na^+^-phosphate buffer). All compounds were added to a final concentration of 10-mM (os  =  optical section; pr  =  projection). Bars are 5 µm in all images.

A fusion protein of the tomato sucrose transporter SlSUT1 with GFP (SlSUT1-GFP) was shown to respond to different treatments with oxidizing compounds (GSSG, L-cystine, H_2_O_2_) with the formation of patchy structures in the yeast plasma membrane [7] that were reminiscent of raft-like microdomains described in yeast [8]. We studied the effect of the reducing compound GSH (Fig. 8C, 8D and 8F) or of the oxidizing compounds GSSG (Fig. 8E) and H_2_O_2_ (Fig. 8G and 8H) on the distribution of GFP-labeled UmSRT1 protein within the plasma membrane of *UmSRT1-GFP*-expressing yeast cells. It has been demonstrated before [1] that UmSRT1-GFP is still a functionally active sucrose transporter.

In contrast to the results obtained with SlSUT1-GFP, we were unable to detect any difference in the distribution of the UmSRT1-GFP-derived fluorescence in the plasma membranes of treated cells and in untreated cells (Fig. 8A and 8B). This result was independent of the use of buffered (Fig. 8C and 8D) or unbuffered solutions (Fig. 8E and 8F) of GSH and GSSG.

In addition to the formation of patchy structures, increased plasma membrane targeting of SlSUT1-GFP has been reported in response to different treatments with oxidizing agents [7]. We found it difficult to make a similar statement for UmSRT1-GFP. In all fluorescent cells, the UmSRT1-GFP-derived fluorescence was primarily in the plasma membrane. The residual fluorescence in endomembranes, however, was variably under all conditions and seemed to correlate with different stages of the cell cycle (Fig. S1).

## Discussion

### pH changes at the plant-fungus interface inversely affect ZmSUT1 and UmSRT1

After the successful infection of maize plants, hyphae of the maize pathogen *U. maydis* preferably grow along the host's phloem vessels, where their sucrose transporter UmSRT1 allows the direct utilization of apoplastic sucrose [1]. This uptake of sucrose by the fungus occurs in competition with the maize sucrose transporter ZmSUT1 that loads sucrose into the phloem [5]. As the activity of plant sucrose transporters, and specifically of ZmSUT1, was shown to respond to protons [6] and redox-active compounds [7], we studied the potential effects of both effectors on sucrose transport proteins acting at the *U. maydis*/maize interface.

Whilst we were unable to confirm the reported effects of redox-active compounds on plant sucrose transporters (Fig. 1 to [Fig pone-0012429-g002] 3 and 6), which is in line with recently published data [10], [12], we pinpointed robust opposite effects of the extracellular pH on the transport activities of fungal and plant sucrose transporter. Lowering the external pH from slightly basic (pH 8.0) to highly acidic values (pH 3.0) caused an initial activation of UmSRT1 with a transport maximum at pH 5 and 6. A further decrease in the apoplastic pH was paralleled by a steep decrease in UmSRT1 activity.

In contrast, plant sucrose transporters are completely or largely inactive at pH 7 and pH 6, partly active (25%) at pH 5, and further activated with decreasing pH values. This was shown in detail in [10] for the potato sucrose transporter StSUT1, and is suggested for the maize sucrose transporter ZmSUT1 by the strong activation of its transport activity at decreased extracellular pH ([10] and present paper; data not shown).

Detailed measurements of apoplastic pH values in plants were performed in several species. In the apoplast of maize roots, pH-values between 5.1 and 5.6 were recorded [13]. In this system, activation or inactivation of the plasma-membrane proton pump caused a decrease to pH 4.8 or an increase to pH 6.2, respectively. Similar values were determined for the apoplast in the elongation zone of roots from lupin (*Lupinus angustifolius*; pH 5.2 to 5.4 [14]), and slightly lower values (pH 4.7 to 5.2) were reported for the apoplast in the substomatal cavity of broad bean (*Vicia faba*) leaves [15]. In this system, the effects of numerous ions, several molecules and physical parameters on the apoplastic pH were determined in detail. Unexpectedly, already minute deviations in the ion composition, in the CO_2_ concentration, or in the light intensity had significant effects on the apoplastic pH [15]. Therefore, apoplastic pH changes were discussed as possible signals for the activation of channel proteins [16], as drought signals [17], [18], or as a signal that can trigger the release of abscisic acid (ABA) into the apoplast [19].

Our data on the pH-dependence of UmSRT1 show that this protein and the corresponding maize sucrose transporter ZmSUT1 respond differentially to changes in the extracellular pH. Interestingly, the fungal sucrose transporter has its pH-optimum in the range thought to be physiological for the plant apoplast, whereas the pH-optimum of plant sucrose transporters lies at much lower pH-values. Changes in the range between pH 4.8 and 6.2, the values obtained in the analyses cited above, would thus significantly affect the still low transport capacity of plant sucrose transporters, but affect the fungal transporter only marginally. Acidification of the apoplast below pH 4.8, would further activate the plant sucrose transporter, but simultaneously reduce the transport capacity of the fungal transporter.

In fact, it has been concluded [15] that apoplastic pH changes can easily be achieved by changes in the transport activity of H^+^-symporters or of the proton pump due to the low passive buffer capacity of the apoplast. Thus, optimization of the apoplastic pH for the specific needs of the host or pathogen sucrose transporter might be a means to adjust the transport capacity of host or pathogen sucrose transporters.

### Redox-active compounds do not alter the subcellular distribution of UmSRT1

It has been suggested [7] that the prominent increase in SUT1-mediated sucrose uptake in yeast upon application of oxidizing agents might be caused by conformational changes of the protein or by differences in the localization. In fact, they could show that 60% of the GFP-labeled SlSUT1 protein was retained intracellularly, and that in the presence of H_2_O_2_, L-cystine, or GSSG most of the initially intracellular SlSUT1-GFP was targeted to the cell surface [7]. Moreover, the treatment with oxidizing compounds caused a relocation of the initially evenly distributed SlSUT1-GFP protein into raft-like structures [8].

When we studied the subcellular distribution of UmSRT1-GFP in response to various redox-active compounds, we observed neither a reproducibly enhanced targeting of UmSRT1-GFP to the plasma membrane and, most importantly, no formation of raft-like structures (Fig. 8). This difference between UmSRT1-GFP and SlSUT1-GFP may, in fact, point towards different responses of these proteins to the treatment with redox-active compounds. However, two different plasma membrane compartments representing non-overlapping, raft-like microdomains were described [20]. These membrane compartments contained either the GFP-labeled plasma membrane ATPase, Pma1p-GFP, or the GFP-labeled arginine/H^+^ symporter, Can1p-GFP. In contrast, Hxt1p, the yeast hexose transporter 1, was not found in any of these microdomains and rather evenly distributed in the yeast plasma membrane [20].

Currently, it is not known, why different yeast proteins reside in specific microdomains of the plasma membrane or why others are evenly distributed. One set of experiments suggests that microdomain-resident proteins are less accessible for internalization and subsequent degradation [21]. The reasons, why foreign proteins, like the hexose/H^+^ symporter from the green alga *Chlorella kessleri*, HUP1, are targeted to a specific plasma membrane microdomain in yeast, is even less obvious. It is discussed that the targeting of HUP1 to a yeast microdomain reflects a similar microdomain localization in *Chlorella* [8], and this is also discussed for the tomato SlSUT1 sucrose/H^+^ symporter [7]. Nevertheless, the reason for the redistribution of SlSUT1 into a plasma membrane microdomain under oxidizing conditions and the continuously even distribution of UmSRT1 under the same conditions is unclear, as the transport activities of both transporters are similarly affected by reducing and oxidizing compounds.

Although the physiological relevance of the observed differences of redox-active compounds on the subcellular targeting of plant and fungal sucrose transporters is unclear, our results clearly demonstrate that extracellular redox changes have no effect on the transport activity of all analyzed sucrose transporters. Most importantly, however, our data suggest that changes in the extracellular pH might be a means for inverse regulation of plant and fungal sucrose transporters at the host/pathogen interface.

We are well aware that as in previous analyses of other groups [7], [12] our data were also obtained exclusively in heterologous expression systems. The hypothesis deduced from analyses in heterologous expression systems will now need to be tested *in planta* to demonstrate the physiological relevance of our data. These *in-planta* analyses of discrete apoplastic pH-changes and of induced modulations of transport activities in individual companion cells of an intact leaf or in single hyphae of a pathogenic fungus will represent a major challenge.

## Materials and Methods

### Yeast strains, transformation and growth conditions

The *UmSRT1*-expressing *Saccharomyces cerevisiae* strain, the corresponding control strain and the *UmSRT1-GFP*-expressing strain were identical to those used in [1]. The *StSUT1*-expressing yeast strain and the corresponding control strain was identical to the strains used in [22] and was originally been obtained by Christina Kühn (Humboldt University of Berlin, Germany). The Δ*hgt1* mutant strain (Y01213) was obtained from BioCat GmbH (Heidelberg, Germany). For the expression of the *StSUT1* cDNA in this strain, the *StSUT1*-encoding plasmid was isolated from the strain published in [22] and transformed into the strain using a published protocol [23]. *Escherichia coli* strain DH5α was used for all cloning steps [24].

### Transport measurements in *S. cerevisiae*

Uptake measurements in *UmSRT1*-expressing or *StSUT1*-expressing *S. cerevisae* cells were performed in 50-mM Na^+^-phosphate buffer (pH 5.5) as described [1]. Uptake experiments were started by adding labeled substrate (^14^C-labeled sucrose; initial concentration 1 mM), redox-active compounds were added 5 min prior to the start of the uptake experiment.

### Measurement in *X. laevis* oocytes

*ZmSUT1* cRNA was prepared using the mMESSAGE mMACHINE™ T7 RNA transcription kit (Ambion Inc., Austin, TX). Oocyte preparation and cRNA injection have been described elsewhere [25]. Two-electrode Voltage Clamp (TEVC) recordings were performed with the use of a TURBO TEC amplifier (NPI Electronic GmbH). The *ZmSUT1*-injected oocytes were perfused with a standard external solution containing 30 mM KCl, 1 mM CaCl_2_, 1 mM MgCl_2_, 80 mM D-sorbitol and 20 mM sucrose based on MES/Tris buffers pH 5.6. All solutions were adjusted to 220 mosmol kg^-1^ using D-sorbitol.

Single recordings were performed at pH 5.6 and a holding potential of −70 mV. Steady state currents were obtained by stepping the membrane potential from the holding potential of −20 mV to a series of 500 ms test pulses from +40 to −130 mV (in 10 mV decrements). Steady state currents in Fig. 6 resemble sucrose-induced currents obtained by the subtraction of currents in the presence of sucrose and the absence of sucrose.

### Quantification of GSH

The stability of GSH was determined with the 5,5′-dithiobis(2-nitrobenzoic acid) (DTNB  =  Ellman's reagent [26], [27]). Analyses were performed according to the manufacturers protocol. The extinction of 2-nitro-5-thiobencoic acid, the cleavage product formed during the reaction of GSH with DTNB, was measured at 412 nm with a NanoDrop® ND-1000 spectrometer (PEQLAB Biotechnologie GmbH, D-91052 Erlangen).

### Subcellular localization of the UmSRT1-GFP fusion protein

The *UmSRT1-GFP*-expressing strain was grown on maltose medium as described [1], harvested at an absorbance at 600 nm (A_600_) of 0.65, washed twice with water and resuspended in 50-mM Na^+^-phosphate buffer pH 5.5 (unless otherwise indicated). Cells were incubated for 30 min at 29°C either without addition of a redox-active compound, or in the presence of 10-mM GSH, GSSG or H_2_O_2_, that were added from 250-mM (GSG and GSSG) or 1-M (H_2_O_2_) stock solutions. Stocks of GSH and GSSG had either been adjusted to pH 5.5 (buffer GSH and GSSG) or not (unbuffered GSH and GSSG).

Confocal images of UmSRT1-GFP in *S. cerevisiae* were determined by confocal microscopy (Leica TCS SPII; Leica Microsystems) and processed with the Leica Confocal Software 2.5 (Leica Microsystems). Emitted fluorescence was monitored at detection wavelengths longer than 510 nm.

## Supporting Information

Figure S1 Intensity and subcellular distribution of GFP fluorescence in UmSRT1-expressing yeast cells is variable. A: Confocal section of untreated UmSRT1-expressing yeast cells. B: Transmission-light image of the cells shown in A. White arrows in A and B identify cells showing no GFP fluorescence. C: Confocal section of untreated UmSRT1-expressing yeast cells. D: Transmission-light image of the cells shown in C. White arrows in C and D identify cells showing no GFP fluorescence, yellow arrows show cells with strong labeling of endomembranes, pink arrows show cells with almost no labeling of endomembranes. E: Confocal section of UmSRT1-expressing yeast cells treated with unbuffered GSH. F: Transmission-light image of the cells shown in E. White arrows in E and F identify cells showing no GFP fluorescence, yellow arrows show cells with strong labeling of endomembranes, pink arrows show cells with almost no labeling of endomembranes. Bars are 10 µm in A to F.
(1.49 MB TIF)
