# Supplementary figures and images for: Inverse pH Regulation of Plant and Fungal Sucrose Transporters: A Mechanism to Regulate Competition for Sucrose at the Host/Pathogen Interface?
Source: PLoS One. 2010 Aug 26;5(8):e12429. doi: 10.1371/journal.pone.0012429 (PMC2928750; doi:10.1371/journal.pone.0012429)

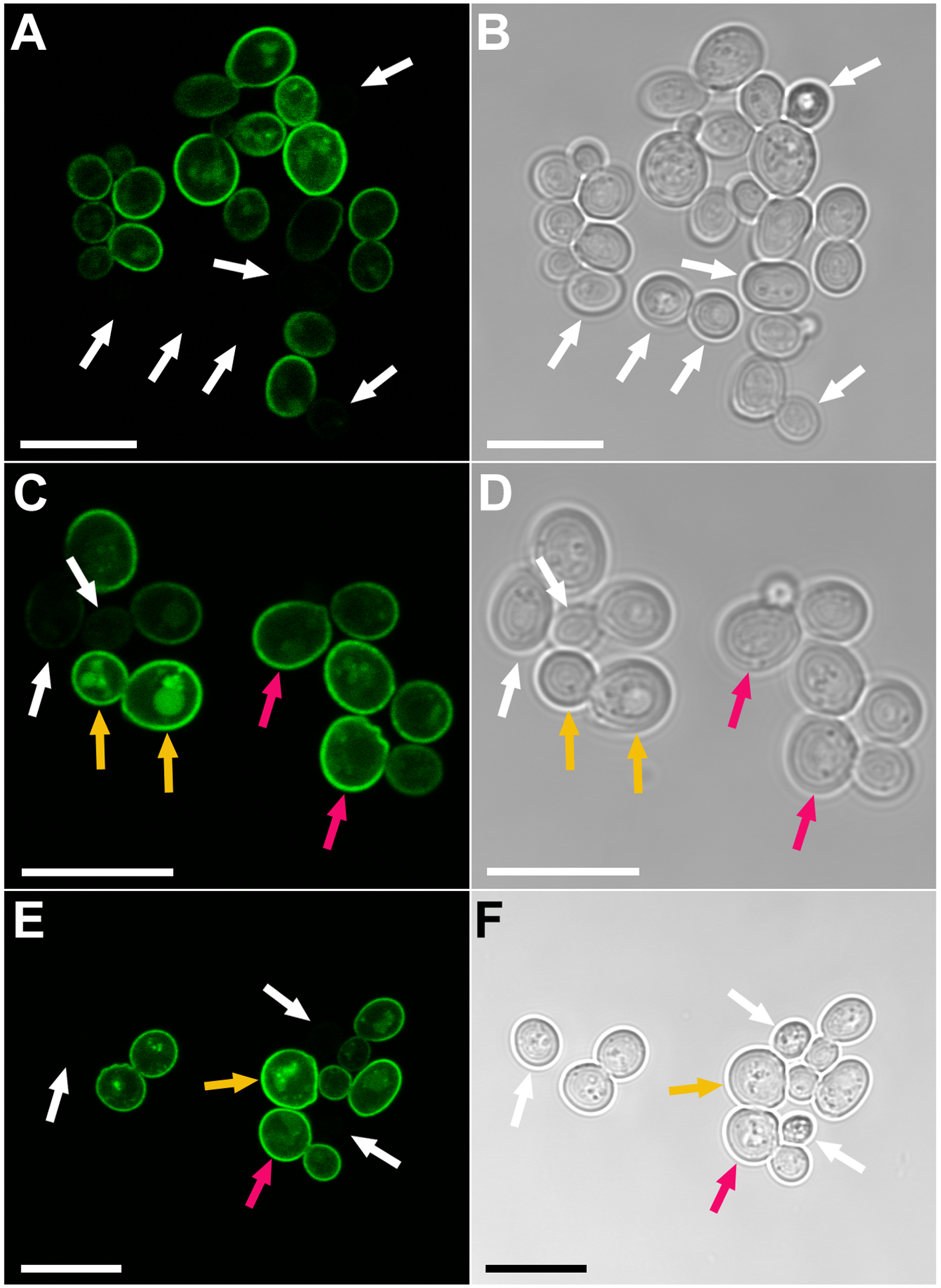

Supplement: Figure S1 — Intensity and subcellular distribution of GFP fluorescence in UmSRT1-expressing yeast cells is variable. A: Confocal section of untreated UmSRT1-expressing yeast cells. B: Transmission-light image of the cells shown in A. White arrows in A and B identify cells showing no GFP fluorescence. C: Confocal section of untreated UmSRT1-expressing yeast cells. D: Transmission-light image of the cells shown in C. White arrows in C and D identify cells showing no GFP fluorescence, yellow arrows show cells with strong labeling of endomembranes, pink arrows show cells with almost no labeling of endomembranes. E: Confocal section of UmSRT1-expressing yeast cells treated with unbuffered GSH. F: Transmission-light image of the cells shown in E. White arrows in E and F identify cells showing no GFP fluorescence, yellow arrows show cells with strong labeling of endomembranes, pink arrows show cells with almost no labeling of endomembranes. Bars are 10 µm in A to F. (1.49 MB TIF) [file pone.0012429.s001.tif]
